# High-Frequency Oscillating Currents

**Published:** 1917-10

**Authors:** John M. Garratt

**Affiliations:** Buffalo, N. Y


### BUFFALO MEDICAL JOURNAL

Yearly Volume 73 OCTOBER, 1917 Number 3

### ORIGINAL ARTICLES

**The right is reserved to decline papers not dealing with practical medical and surgical subjects, and such as might offend or fail to interest readers. Contributors are solely responsible for opinions, methods of expression and revision of proof.**

### High-Frequency Oscillating Currents.

By JOHN M. GARRATT, M. D., of Buffalo, N. Y.

In the Journal of the American Medical Association we find the following statement: "The high-frequency currents are understood by few, misunderstood by many, and underestimated by everybody."

Misunderstandings are usually brought about by the use of inappropriate apparatus or technique with consequent failure to produce certain changes in the tissues necessary before relief of a pathological condition can be accomplished.

It is hoped that this article will aid in giving a better understanding of these currents and will also help to clear up misunderstandings.

It is a universally conceded fact that the high-frequency currents, when properly applied, do exert a most wonderful and peculiar effect upon the human body.

#### DISCOVERY AND DEVELOPMENT OF THE HIGH-FREQUENCY CURRENTS.

The most important part of the apparatus for the production of high-frequency currents, is the condenser or Leyden jar. This was discovered by Cuneus in 1745 and was named after the city where it was first used.

In 1881 Dr. W. J. Morton of New York produced his "Static Induced Current"—the basic arrangement of all high-frequency currents.

Ten years later Nicola Tesla demonstrated by a series of brilliant experiments, his "Alternate Currents of High-potential and High-frequency" before the American Institute of Electrical Engineers. He lighted lamps with currents passing harmlessly through the body and heated wire to incandescence by the touch of his conducting hand; apparently defying death. Lately he has caused sparks to leap an air gap twenty-five feet in length and two or three feet in diameter. The voltage was in the billions and the amperage 800.

The development of the high-frequency currents, are mainly due to the efforts of Tesla and Elihu Thompson, American scientists. Their experiments were confined to the bipolar high potential currents.

D'Arsonval of France developed for therapeutic use the low potential currents. Oudin also of France the monopolar high potential currents.

CHOICE OF APPARATUS; TESLA APPARATUS—consists of a specially constructed transformer energized by an alternating current. From the secondary terminals one leads to the inner armature of a Leyden jar, the outer armature of which is connected to one terminal of the primary of the Tesla coil; the second, broken by a spark gap, leads to the other terminal of the Tesla coil. This coil is made up of a primary with a few turns of coarse wire and a secondary of a greater number of turns of fine wire. The oscillations obtained from a Tesla apparatus is fabulously high—millions a second and the potential may be hundreds of thousands or millions of volts. The brush discharge (effluve) may be 20 or even 40 inches in length. There are many modifications of this apparatus.

D'ARSONVAL APPARATUS:—D'Arsonval conceived the idea of using a solenoid of copper, for the alternative path of the current between the external armatures of two Leyden jars. He introduced this arrangement in 1890. The inner armatures are connected to the terminals of any apparatus which will supply a high potential current—a static machine, transformer or Rhumkorff coil. The one used, depending upon the current supply and the effects desired. The outer armatures of the Leyden jars are short circuited through a solenoid of coarse wire which induces a considerable self-inductance. A spark gap is placed between the inner terminals of the Leyden jars for the purpose of disrupting the current. This spark gap may be single or multiple; the latter increases its effect.

The writer has designed and constructed an apparatus, which for vacuum tube and autoconduction treatment, leaves little to be desired. The source of the high potential current is a 14 inch induction coil specially wound for voltage—the secondary containing 172,000 feet of No. 36 D. S. C. magnet wire. The secondary discharges into condensers of large capacity, the external armatures are short circuited through a few turns of very coarse aluminum wire to form a solenoid 22″ in diameter, from which is derived the d'Arsonval current. In the center of the solenoid is fixed a drum 12″ in diameter and 30″ in length upon which is wound about 500 turns of wire, from this coil is obtained Tesla currents and it gives an effluve which will leap 14″.

OUDIN APPARATUS:—differs from the d'Arsonval in the solenoid which is built up with 30 to 100 turns of smaller wire and the upper connection from one Leyden jar is arranged so that it can be adjusted from one turn of wire to another for the purpose of tuning. When properly in tune the upper part of the solenoid resonates in unison with the lower as would a tuning fork resonate with another of the same pitch.

To understand the action of the high-frequency current it is necessary to have a knowledge of the laws of induction and the phenomena of Leyden jar discharges.

PHYSICAL PROPERTIES OF THE HIGH-FREQUENCY CURRENTS:

(1) **Induction Effects** are due to the action of an electro magnetic flux on a neighboring susceptible body to the point of induced magnetic saturation. The degree or intensity of the E. M. F. is proportionate to that of the rate of variation of the magnetic charges multiplied by the frequency. One may produce an effect of high-frequency and low tension equal to that of a high tension and low frequency in the same mass.

(2) **Electro Static Properties** are physical phenomena analogous to that produced by the influence or static machine of the Wimshurst type. The electro static field is shown by: (a) The glow of vaccum tubes when brought near but not touching the terminals. (b) When two wires connected one to each terminal of a high-frequency apparatus, are held parallel to each other in the dark, sparks are seen leaping from one to the other. (c) A collection of particles of carbon or metalic dust or similar matter on the generator. (d) The generation of ozone. (e) Effluve.

(3) **Dyanamic Properties** are shown when the two terminals of the machine are connected with a very fine wire of high resistance; when the current is turned on the wire will glow and fuse from overload.

(4) **Resonancy**—See explanation under Oudin apparatus.

THE PHENOMENA OF LEYDEN JAR DISCHARGES—Leyden jar discharges are of an oscillating nature. The flow of the current is periodically reversed and the opposite wave length decreases successively in the same ratio—this it does millions of times a second. Sir Oliver Lodge named the discharge from the internal armature the ''A'' spark and that from the external armature the ''B'' spark. The ''A'' spark is oscillatory with the plus in excess in one direction and if it is applied to the tissues an electrolizing action is demonstrated by the destruction of the tissue. The ''B'' spark is oscillatory but with the wave-length equal in both directions; it is therefore harmless—there is no electrolizing action.

### EFFLUVE AND VACUUM TUBE CURRENTS.

From the terminal of an active Oudin or Tesla coil, is seen a beautiful brush-like discharge (effluve) of bluish color which will leap to any object brought near it. If the object is a piece of glass, one-half inch thick or more, it is apparently penetrated by the currents. The electric waves are transmitted through the air with almost the velocity of light waves which travel at something like 186,000 miles a second.

Vacuum tubes are excited by the Oudin and Tesla currents. The tubes are made in a variety of shapes to facilitate the application of the currents to the skin or cavities of the body. The effects produced by vacuum tube currents are said to differ with the degree of vacuum: 1-500 of an atmosphere gives the active tube a pinkish color and the application is less irritating—indicated in acute localized lesions. A still higher vacuum gives to the tube a bluish color and if the exhaustion is carried to 1-2000 of an atmosphere a whitish luminosity is produced—indicated in chronic conditions. With a vacuum of 1-100,000 or over X-rays are generated in addition to the electrical discharges.

Vacuum tube discharges are in effect nebulized streams of particles, which Sir Oliver Lodge asserts are composed of electrons which pass through the glass and if sufficiently accelerated, excite radiation.

THE EFFECTS OF EFFLUVATION AND VACUUM DISCHARGES are due to:

1—Electrical oscillations which relieve congestion, stimulate nutrition, and increase the activity of the vasomotor and trophic nerves.

2—Ionic bombardment which is a stimulating counter-irritant.

3—Liberation of ozone which is a powerful oxidant, disinfectant and antiseptic.

The action of nitric oxid, light and heat waves and Hertizian radiation undoubtedly contribute to the effects.

The effluve applied directly to the skin is especially valuable in septic conditions, unhealthy scar tissues, etc. The growth of pus forming organisms are inhibited and their toxins powerfully modified.

VACUUM TUBE DISCHARGES are usually applied directly to the patient. The application is called **stabile** when the electrode is applied firmly to one spot or **labile** when the electrode is constantly moved over the surface treated. When the active tube is in contact with the skin, a sedative effect is produced. A spray of sparks stimulates, and undoubtedly promotes absorption of inflammatory exudates and adhesions. The applications cause a hyperaemia of the skin and exerts a beneficial action in painful conditions as neuritis, neuralgia, rheumatism, laryngitis, etc. Sparks applied to the spine will raise arterial tension.

FULGURATION or lightening treatment consists of the application of high-frequency sparks to the tissues of the body by means of a metal electrode, for the purpose of:

1—**Dehydration**: An effect produced by the application of sparks from a current of high voltage and low amperage, as from a Tesla coil, Oudin resonator of static machine. Also called "Cold spark fulguration."

2—**Cauterization** obtained from the low voltage high amperage currents delivered by the d'Arsonval solenoid—Hot spark fulguration.

There are two methods of applying fulguration:

1—**Direct Method**—Delivers the sparks from the apparatus to the patient.

2—**Indirect Method**—The patient is connected to the apparatus by a metal electrode, the fulguration electrode being grounded.

Fulguration is useful in removing small growths, warts, moles, and papillomata—from the skin and mucous membranes.

Fulguration was introduced by DeKeating-Hart of Marseilles. He used the Oudin resonator and applied sparks with a special electrode which he devised. In addition to the metal used to carry the current, is a tube through which air or CO2 is forced. The procedure is both surgical and electrical since the sparks alone would require too many and too long exposures, and the elimination of toxic products might lead to dangerous consequences. So surgical interference must always precede the electrical treatment. Following this treatment there is a lymphorrhoea which assists in draining the lymphatics of migratory cells and at the same time myriads of beneficial leucocytes are brought to the diseased area.

Fulguration according to Hugh Young is superior to any other method for the cure of papilloma of the bladder which only a few years ago many authorities considered almost impossible to cure. He says: "This demonstration of the marvelous efficiency of the high-frequency currents is one of the most brilliant and valuable additions to surgery in recent years."

HIGH-FREQUENCY WAVE CURRENTS—They are obtained directly from the external armatures of the condensers. One electrode may be of metal and the other a vacuum tube; or, both electrodes may be of metal in which case one is insulated from the patient. Its action is that produced by the Faradic Current plus high-frequency oscillations. The action upon the motor nerves and muscular tissues can be regulated from mild to intense reactions.

These currents are indicated in certain forms of prostatic hypertrophy, seminal vesiculitis, and relaxed mucous membranes, paralysis, etc.

DIATHERMY AND THERMOPENETRATION are synonymous terms which apply to high-frequency currents of low voltage and high amperage when used to generate heat in the tissues through which the current surges.

Nagelschmidt, in 1907, demonstrated and recommended thermopenetration in the treatment of diseases of the joints and circulation. At a congress in Budapest in 1908, he brought out his improved apparatus designed especially for "diathermy." He found that the apparatus in use at the time did not produce sufficient amperage because the oscillations were not sustained; especially was this the case when an induction coil was used to charge the condensers:—it was determined that they discharged during a period of 1/5th of a second and that in the interval of 4/5ths of a second no current passed. The periods of discharge can be calculated from (a) the capacity of the condensers and (b) the resistance of the circuit and its co-efficient of self inductance. For efficient apparatus certain values must be up to a standard.

The heat developed is governed by the strength of the current; the rapidity of the oscillations and the resistance in the circuit opposing its flow.

The heat produced is unique in that little or none is felt at the point of contact—it is greatest midway between the electrodes, providing they are of equal size. This is a very important effect and permits of localized action upon the deeper tissues.

MEDICAL DIATHERMY has to do with a moderate rise of temperature in the tissues when the currents are applied locally or generally. It supplies heat to the part and in the quantity desired and increases instead of exhausting the energy of the living cells. It produces general pyrexia-phisiological fever—Nature's first line of defence against the invasion of pathologic bacteria—without the production of toxins.

In rheumatism and gout it is the ideal treatment; it relaxes arterial spasm, gives relief from pain and swelling and by the secondary hyperaemia produced promotes phagocytosis and improves metabolism.

Gonococci in the tissues are destroyed by a temperature of 41 C. which is easily produced by diathermy.

Recent observations have shown that female patients suffering from exhausting discharges have speedily regained their health as a result of diathermic stimulation of the pelvic tissues.

SURGICAL DIATHERMY or electrical coagulation has lately been used extensively by Doyen for the destruction of neoplasms. To quote DeKraft "The method has given beautiful results—it sterilizes while it destroys, opens no vessels, nor does it cause proximate or distant metastases. Cancer cells are destroyed at 55 C. They lose their virulence at 50 C. Healthy cells are destroyed at 60 C. Burning of the second degree takes place at 70 C. and the third degree at 75 C."

The indifferent electrode should be of large surface; the active is usually one or more needles which are inserted into the mass to be destroyed.

Experiment 1—Place two electrodes of equal size into a solution of egg albumen and turn on the current gradually. Coagulation will first appear midway between the electrodes.

Experiment 2—Repeat, but turn the current on suddenly full strength. The coagulation will appear first at the electrodes.

In both experiments if the current is allowed to surge for a sufficient time the coagulation will completely fill the space between the electrodes.

Experiment 3—Two disk electrodes of equal surface are placed on opposite sides of a potato. Allow one ampere of current to surge for a minute or two. The potato starch will be cooked in the center and will respond to the KI test while the starch at the periphery will be unchanged.

Experiment 4—Use two disk electrodes of one inch surface. Place on opposite surfaces of a two inch cube of raw meat; using the same current as above. Coagulation proteid will fill the spaces between the disks.

Maragiano placed a small electric lamp in the thorax of a dog. Two metal plates were placed one on each opposite pleura. When the current was turned on, the filament of the lamp became incandescent, showing the penetrating effect of the current.

AUTO-CONDUCTION AND AUTO-CONDENSATION — Auto-conduction or d'Arsonval's method. Any conductor placed within and enveloping high-frequency circuit, becomes the seat of currents of auto-conduction similar to Foucault currents. The patient is placed within a horizontal or vertical solenoid which may encircle a couch or arbor (original). The patient is not in actual contact with the solenoid. The currents may be demonstrated by connecting a 20 V. incandescent lamp with three turns of heavily insulated No. 14 wire and placing it within the solenoid so that the two windings are parallel to each other. When the current is turned on the filament of the lamp will become incandescent.

In Auto-condensation, the body of the patient is in direct contact with one terminal of the d'Arsonval solenoid; the other terminal is connected to a large metal plate, insulated from the patient placed on a couch or chair. The precise interpretation of the phenomena of high-frequency condensation is not as yet satisfactorily explained, but the accepted explanation is that the patient's body becomes one armature of a condenser.

In the reduction of high blood pressure auto-condensation gives us the best and most lasting results and without cardiac depression.

Dr. Samuel Sloan of Glasgow, reported in the London Lancet, June 1907, the result of his extensive investigations as to the effect of the high-frequency currents upon the heart and blood pressure. Some of his conclusions follow:

1—"These currents cause at first, in all cases, diminished peripheral resistance.

2—"In all cases this is followed sooner or later by increased cardiac force when the current is given in therapeutic doses.

3—"The effects on the blood pressure and the pulse-rate of this double action will depend on the cardio-vascular stability.

4—"Should this be normal there will be no change whatever save an increase in blood flow giving rise to a slight elevation of the temperature of the blood.

5—"When the blood pressure is high for the individual's age and there is no apparent disease to account for it, I have observed that after several applications of the current the blood pressure is diminished and there is a corresponding improvement in the pulse rate.

6—"Upon rhythm the effects of electrification are marked. In some instances of intermittent pulse—radial only, or cardiac as well—the intermissions will diminish or disappear, even in a case which had caused anxiety to the patient for many years. If the intermissions are recent, as from temporary digestive disturbance, they may cease after only two or three electrifications, along with the associated benefit of the current on the gastro-intestinal canal.

7—"When the pulse is variable before electrification it may become quite regular and steady immediately after.

8—"These researches offer sufficient evidence to establish my contention as to the tonic effects of high-frequency currents on the heart, a subject which is of the greatest importance."

Under auto-conduction and auto-condensation competent observers tell us that tubercular areas produced in healthy guinea pigs heal, leaving healthy scar tissue. Applied to human beings favorable changes take place. In general nutrition there is an increase in the arterial circulation and in the number of respirations; there is an increased diuresis and a better elimination of the excreta; there is an increase in organic combustion; the disproportions between uric acid and urea are brought to the normal ratio (1:40). The amount of oxygen in the blood is increased and may even exceed the normal physiologic limit of six per cent. Patients under treatment exhibit a better capacity for work and walking. Flabby muscles become firm. Apostoli reporting the results of treatment in 518 cases noted improvement in general health, increased energy, return of appetite, better sleep, improved digestion and disposition in patients with bad tempers. He claims it "a medicament for the cells and a powerful modifier of the general nutrition which it can at once promote and regulate.

REPORTS OF CASES.

Case 1. NEURITIS—The results accomplished in neuritis are best described in the following letter received from a grateful patient and printed in part with his permission.

"About nine years ago, I injured my right arm while alighting from a train, simply a slight blow on the elbow which resulted in a Traumatic Neuritis. The thumb, first two fingers and part of the arm were paralized for some five or six weeks. Since that time, the use of my hand and arm has been more or less restricted. Should I use my arm to any great extent it would result in severe pain, which generally seemed to increase at night or when lying down.

"I have tried various treatments such as baking with a Kelley heater, massage, hot packs, also electric treatments, but the results were not at all satisfactory as the pain continued; my hand would become numb and at times the fingers would cramp badly. During September and October (1913)

the pain became unusually severe and on October 24th at the suggestion of Dr. Schreiner I called to see you.

"I received thirty-three high-frequency treatments. After the first treatment the pain increased greatly, but the second produced more satisfactory results. After the third and fourth there was a very marked change, and while at times the pain entirely left me, I did at times have considerable pain. After the fourteenth treatment the arm improved rapidly. Since the first of the year (1914) there has been no recurrence of the pain, numbness, or cramps, and I have used my arm freely."

Case 2. INFECTION—Dr. S. infected right thumb of three months duration, two operations had been performed under nitrous oxid anesthesia. A sinus persisted from which pus discharged necessitating redressing twice daily. The thumb was swollen and painful and gradually getting worse so that he was becoming alarmed about it. A Roentgenogram showed involvement of the terminal phalanx.

Three applications with effluvation checked the discharge and the pain and swelling was almost nil. After the seventh application the sinus had closed and a cure affected.

Case 3. CHRONIC ARTICULAR RHEUMATISM—Female, aged 45. Referred by Dr. C. F. Howard. At the age of 15 she strained the right shoulder while lifting an invalid sister. It was painful thereafter for five years off and on.

The present trouble was first noticed in the fall of 1914. It commenced suddenly with pain in the right shoulder, while carrying a tray of dishes; it gradually increased so that at time of entry, April 3d, 1915, it was at times very severe, especially at night and in damp, stormy weather. Voluntary motion of the arm was almost completely lost and passive motion showed apparent ankylosis of the shoulder joint. Blisters and drugs had been tried by another physician with no improvement. A Roentgenogram of the shoulder showed a marked osteoporosis of all the shoulder bones; there was no apparent erosion of the articulating surfaces.

There was no fever or swelling in the joint at any time and no other joint was involved. There was some muscular atrophy from disuse. Hand dynamometer reading—right 15°, left 60°.

Twenty-two high-frequency treatments (auto-condensation and vacuum currents) were given, the last on June 23, '15, at which time pain had disappeared, motion was re-established so that she could comb her hair. Hand dynometer reading—right 52°, left 80°. General health had greatly improved and weight increased.

Case 4. Male, aged 48. Referred by Dr. Jas. Croff.

His foot was injured on June 16th, '16, by a discharge of dynamite which went off under a plank upon which the foot was resting. On July 28th the soft parts of the foot were considerably swollen and congested, he could not bear even a small part of his weight upon it; there was loss of sensation in the heel.

Roentgen findings: A vertical fracture through the external border of the astragalus; comminuted fracture of the os calcis with 1/8th inch upward displacement of the fragment and longitudinal splintering of the inferior surface.

Treatment consisted of auto-condensation to improve metabolism and the application of the vacuum electrode to the foot. Improvement was noted from the first; after the third swelling had nearly disappeared; sensation was returning to heel; after the eighth he could wear his shoe and after the 23rd treatment he could walk short distances without support.

Case 5. PAINFUL SCAR TISSUE, NEURASTHENIA. Female, aged 32. Office work. Referred by Dr. Hendee.

**Symptoms**—Lack of energy, easily tired, depressed, insomnia, constipation, cold extremities. Areas of tenderness along the spine, violent pulsation of the abdominal aorta.

Following an operation upon the right kidney five years ago the scar had been more or less painful, especially the later part of the day.

**Treatment** consisted of auto-condensation and the application of the vacuum currents to the spine, abdomen and scar.

Progress—After the second treatment sleep was nearly normal; after the tenth, pain in the scar had disappeared as had also the tender areas along the spine. She was stronger and had more ambition and better spirits. Had to work from 8 a. m. to 9 p. m. "Stood it fine. Previously all in at 3 p. m."

Case 6. PROSTATIC HYPERTROPHY—The results accomplished are described in a letter received from a patient:

"My trouble, which must have been of long standing did not culminate into a serious condition until the spring and summer of 1913. I was reduced in weight 20 lbs. My rest at night was so disturbed that sleep was impossible. The frequency of urination would be every five or ten minutes, accompanied with considerable pain. There were nights when relief was obtained only by sending for a nurse and the use of the catheter. Finally I consulted with our family physician, Dr. Hoag, my first personal experience with a doctor in my life, and I am 63 years of age. The doctor at once diagnosed my case as prostatic enlargement and told me it was either electricity or the knife, and the former was preferable. During the first month's treatment the catheter was used a few times but the necessity decreased as the treatment progressed. By the 1st of the second month the improvement was so marked that I was rarely disturbed at night more than once. Feeling quite myself again I took but six treatments the third month and now my condition is so nearly normal that I am not conscious of my physical ailment. My gratitude is expressible."

---

**Eye Injuries in Wars.** Bourdier, Le Progres Med., May 5, gives the following statistics: U. S. Civil War, total 1190, 0.5% of all wounds, 5.5% of wounds of head; Crimean War, 595; Italian War 97; Franco-German War, 1870, French statistics, 860, 0.86, 8.5%; Russo-Turkish War, 290, 2.21%, 18%; Graeco-Turco-Bulgarian War, 118, 0.85% of total wounds. It appears, therefore that the number of eye injuries has varied from 0.5% to nearly 3% of all wounds in previous wars and that wounds of the head comprise about 10% of all wounds. However, as the proportion of head wounds increases, those of the eyes do not increase proportionately. It is too soon to give anything like accurate statistics for the present war but it appears that the proportion of head wounds is not so great as might be expected from the greatly increased use of trenches, partly because even trench warfare involves a considerable amount of fighting in the open in attacks, partly because of the use of protective helmets, periscopes, etc., partly because, as compared with previous wars, even wounds received in trenches are largely due to artillery, grenades, etc., that is to so massive explosions that the effects are not limited to theoretically exposed, upper parts of the body. Thus de St. Martin observed 73 ocular lesions, comprising 1.36% of the total wounded; Genet, 82, comprising 2.4% of the total; the percentage varying from 0.2% for open fighting to 5.7% for trench warfare. The author's statistics at a hospital of evacuation at Verdun were 5.14%. Up to Dec. 1, 1916, the author saw at a single station, 1021 eye injuries, more than occurred in the entire War of 1870. For the nine months, Mch.-Nov. 1916, the author saw 238 cases of perforation of the globe, 132 due to war wounds, 106 to accidents. In the same period, loss of vision by destruction of the globe was noted in 71 cases, of which 8 were bilateral (total blindness).

---

**Extraction of Intra-Pulmonary Foreign Bodies.** J. Guyot, Paris Medical, Apr. 9, 1917, gives the general impression that extraction by forceps, before the X-ray screen, without anaesthesia, is simpler and more successful than is usually held.

